# Maintenance ramucirumab monotherapy after intolerable toxicities following docetaxel plus ramucirumab

**DOI:** 10.18632/oncotarget.25623

**Published:** 2018-06-19

**Authors:** Akito Hata, Nobuyuki Katakami

**Affiliations:** Akito Hata: Department of Medical Oncology, Kobe City Medical Center General Hospital, Kobe, Japan

**Keywords:** maintenance, ramucirumab, docetaxel, toxicities

We recently reported the efficacy and safety of docetaxel (DOC) plus ramucirumab (RAM) with primary prophylactic pegylated (PEG)-granulocyte-colony stimulating factor (G-CSF) for pretreated non-small cell lung cancer (NSCLC) [1]. DOC+RAM exhibited high response and disease control rates as a salvage-line chemotherapy [2, 3]. However, some cases receiving this regimen suffered from intolerable toxicities such as oral mucositis, anorexia, malaise, nail change, and/or peripheral edema. Even if clinical benefit is obtained, such intolerable toxicities occasionally make it difficult to continue DOC+RAM therapy. In such cases, we actively adopt maintenance RAM monotherapy without DOC.

**Figure 1:**
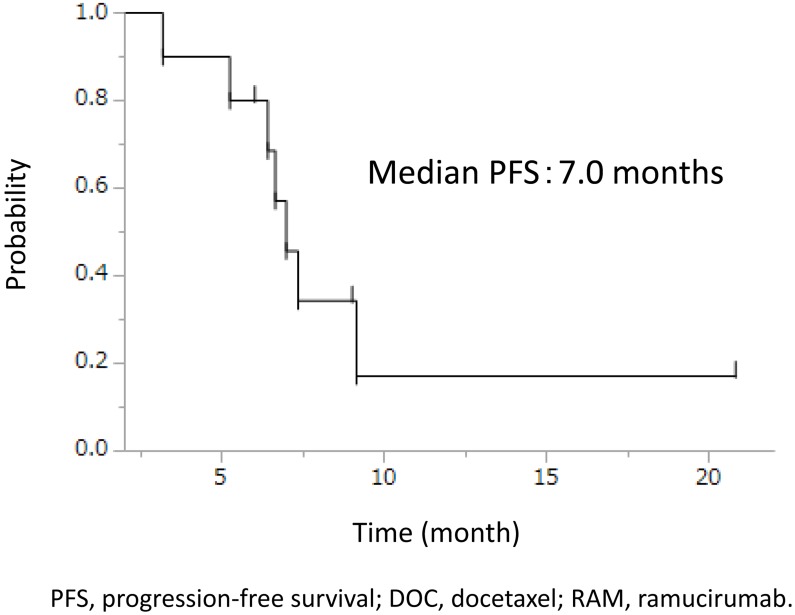
PFS from DOC+RAM initiation

**Figure 2:**
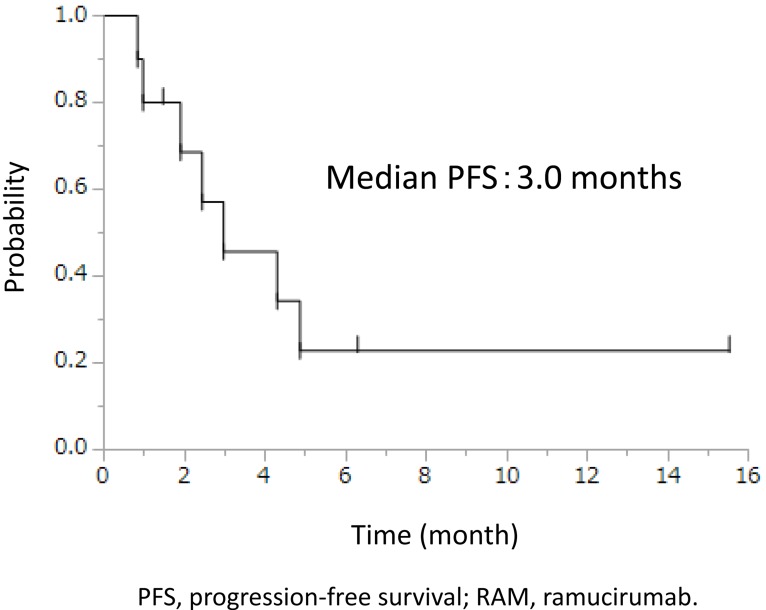
PFS of RAM monotherapy

In our cohort, 10 (19%) of 52 patients receiving DOC+RAM with PEG-G-CSF moved to maintenance RAM monotherapy after intolerable toxicities following clinical response (1 complete response, 5 partial response, and 4 stable disease). Reasons for these movements were all adverse events (AEs) ≥grade 2: 4 oral mucositis; 3 anorexia; 2 malaise; 2 numbness; 2 nail change; and 2 peripheral edema (some overlapping). Median cycles of DOC+RAM before RAM monotherapy was 6 (range, 3-6). Median cycles of RAM monotherapy was 4 (range, 1-19). Among these 10 patients, median progression-free survival (PFS) from DOC+RAM initiation was 7.0 (range, 3.2-21.0+) months (Figure 1), and that of RAM monotherapy was 3.0 (range, 0.8-15.5+) months (Figure 2). Median overall survival (OS) was not reached. All intolerable toxicities during DOC+RAM were improved after moving to RAM monotherapy. Three (30%) of the 10 patients are on treatment without progression. AEs observed during RAM monotherapy were 4 grade 2 hypertension and 1 grade 3 proteinuria, representing an extremely high tolerability.

To the best of our knowledge, this is the first report to suggest the effectiveness of maintenance RAM monotherapy following DOC+RAM in pretreated NSCLC. Ramucirumab is a recombinant monoclonal antibody of the IgG1 class that binds to vascular endothelial growth factor receptor-2 (VEGFR-2) and blocks the activation of the receptor, preventing tumor angiogenesis, growth, and proliferation. In salvage-line settings of gastric cancer, RAM monotherapy demonstrated survival benefit over placebo [4]. This evidence implies prognostic contribution by RAM monotherapy for advanced cancers. Similar results for another VEGF inhibitor, bevacizumab (BEV) were reported in a pivotal phase III study. The E4599 trial comparing carboplatin plus paclitaxel with or without BEV for non-squamous NSCLC showed superior RR, PFS, and OS in the triple combination therapy [5]. In the study, maintenance BEV monotherapy was continued after induction triple combination therapy. This maintenance BEV monotherapy might have affected prolonged PFS and OS, while reducing toxicities.

In patients suffering from intolerable toxicities following response to DOC+RAM, movement to RAM monotherapy could reduce severe AEs, improve quality of life, and prolong PFS. Further studies are warranted to evaluate this unique strategy of maintenance RAM monotherapy in pretreated NSCLC.
